# 

**Published:** 1857-09

**Authors:** 


### INDEX TO VOLUME III.

PAGE.

Asclepias Incarnata,......727
A Case of Menorrhagia. By Moyer,...... 16
Arsenic and Stillingia,......53
Additional Observations upon Verat. Viride. By V. H. Taliaferro,...... 86
A Case of loss of part of the Bones of the Skull,......104
A Thesis on Ves. Vag.—Fistula. By C. Hilliard,......129
Asiatic Chelera and Cholera Morbus,......170
Andrews on Physiology, &c.,......177
Address—Introductory, &c. By W. F. Westmoreland,......579
Abuse of the Pessary. By J. S. Weatherly,...590
Atlanta Medical College,......658
Annual Address before State Society,......570
Adaptation of Sutures,......479
Atlanta Medical College,......505
A Remarkable Case,......510
Alcoholic Stimulants in Snake-bites,......762
American Medical Association...... 382, 55, 126, 513, 657, 660, 312, 762
Adhesions of Placenta By J. W. Jones, sen. 385
A merited Compliment,......377
Artificial Rupture of Amniotic Sac, &c.,......291
Asylum for Idiots......314
Attempted Abortion by American Hellebore, 320
Atlanta Medical College,......247
A School of Medicine at Algiers,......233
Bellevue Hospital, ......128
Bibliographical,......659, 568, 293
Belladonna in Hernia,......315
Books Received,......706
Belladonna in Hooping Cough,......127
Chloroform. By J. S. Weatherly,...... 21
Close of the Volume,......760
Close of Session, ...... 59
Consolidation of Medical Schools,...... 60
Cholera Infantum,......744
Commencement Exercises,......125
Cold Applications in Pleurisy, &c.,......191
Complicated Case of Obstetrics. By B. T. Smith,......593
Chlorate of Potassa. By J. S. Weatherly,......315
Cases of Heroic Doses of Calomel. By Wm. H user,......518
Course of Lectures for 1857,......568
Chlorate of Potassa in Typhoid Fever, ......446
Chair of Chemistry, &c., in Atlanta Medical College,......377
Croup,......755
Cesarean Operation, &c.,......316
Changeability of Disease,......707
Dr. Bowlihg,......210
Difficulties of Diagnosis,......723
Dyspnea, ......317
Difficulties in Diagnosis of Pneumonia,......428
Death of Charlotte Bronte,......188
Dysentery,......62
Electricity, &c., in Suppression of Lac. Secretion,...... 63
Editorial Responsibility,......189
Effects of Decayed Teeth. By J. S. Dixon,.... 89
Extracts, &c., of Atlanta Medical Society. By V. H. Taliaferro,......457
Ethnology of the Negro, &c.,;......464
Extracts, &c., of Boston Medical Society,......491
Effects of Fear,......514
Erratum,......514
Experimental Physiology,......441
Elephantiasis Arabum, ......374
Extracts from Baltimore Pathological Society,......549
Endowment of Atlanta Medical College,......310
Early Operation for Hare-Lip,......316
Extirpation of Clavicle,......318
Ergot in Menorrhagia,...... 60
Ergotine in Diarrhœa...... 63
Fracture of Neck of Humerus, ......486
First Medical Prize,...... 59
Gelseminum Sempervirens, ...... 60
Hemorrhoids,......318
Healthfulness of Atlanta. By F. D. Thurman, 648
Iodide of Calomel, ...... 62
Interesting to Physicians,...... 62
Illustrations, &c., between Uterus and Bladder,......687
Influence of Anatomy, &c.,......339
Immoral Advertisements,......379
Intestinal Animalculæ,......383
Inflammation, &c., of the Cervix Uuteri. By V. H. Taliaferro,......257
Iodine, &c., in Uterine Hemorrhage,......253
Journal of Materia Medica, &c.,......577
Jefferson Medical College,......445
Letters from Paris,......236
Ligature of Illiac Artery and Vein,......317
Lecture on Nerves and Heart,......353
Lead Colic,......442
Lecture on Cancer Cures, &c.,......161
Meterological Table,......128, 89
Mechanism of Nervous Action,......150
Medical Societies. &c.,......431, 523
Medical and Surgical Reports,......570
Medical College of S. C.,......445
Museum of the Atlanta Medical College,......761
Medical College of Georgia,......571
Meteorology Collateral to Medicine. By E. M. Pendleton,......321
Medical Classes in St. Louis,......310
Medical Schools,......231
Medical College of Georgia,......125
Medical Society—present Session,......253
Morbid Effects of Diseased Teeth. By F. D. Thurman,......200
New York Academy of Medicine,......253
Nitras Argent in Tooth-ache,......320
" " " " " By J. S. Dixon, 398
New Mode of Applying Actual Cautery,......513
North American Medico-Chirurgical Review, 445
Notes on Hospitals of Paris,......653
Newspaper Puffing, &c.,......512
New York Lunatic Asylum,...... 60
On Belladonna. By J. H. Connaha,...... 24
On the prevention of Abscess,...... 91
On the Injection of Urea in the Blood,......560
Oglethorpe Medical and Surgical Journal,......571
Observations upon Otorrhœa,......506
On Substances introduced into the Blood,......623
Oglethorpe Medical College,......511
Observations upon Audition. By A. M. Parker,327
Our Journal,......377
On Pathology of Leucorrhœa,......212
On the use of Aconite,......254
Pathology of Hysteria. By N. B. Drurey,......274
Psychology of Acute Rheumatism......277
Prolapsus Uteri. By J. G. Westmoreland......336
Puerperal Fever,......359
Prof. A. H. Buchanan's Address......378
Potassæ Chloras in Typhoid Fever. By V. H. Taliaferro,......389
Prof. Means,......449
Payments for the Journal,......450
Puerperal Convulsions,......495
Paris Hospitals,......578
Proceedings of Montgomery County Society,.670
Preparatory Medical School,......703
Pulmonary Consumption,......108
Professional Secrecy,......156
Paris Correspondence,...... 1
Pork and Potatoes,...... 49
Preservations of Vaccine Virus,...... 61
Prospectus of Savannah Journal of Med.,......508
Prof. Dan'l F. Wright, M. D.,......762
Prizes of the Massachusetts Med. Society......770
Quackery Rampant,......319

PAGE.
Remarks upon Ciliary Blepharitis. By W. F. Westmoreland............................................193
Reproduction of Bones,............................................228
Revaccination,............................................316
Remarks on Syphilization,............................................383
Resection of the Superior Maxillary. By W. F. Westmoreland,............................................393
Remarks upon Inflammation, &c. By F. D. Thurman,............................................454
Rights of Authors,............................................509
Receipts,............................................672, 514
Removal of Urinary Calculi,............................................606
Report of the State Society of Michigan, &c...614
571 Report to the State Society, &c.,............................................[illegible]
Report of a Case of Monstrosity. By J. E. G. Terrill,............................................651
Retention of Urine, &c. By W. A. Love,........652
Remarks on Moral Insanity, &c............................................678
Report of Committee on Medical Education,..697
Review of Nature and Art in the Cure of Disease,............................................33
Something "New under the Sun." By J. Boring,............................................19
Sudden Deaths in the Puerperal State,........... 25
Singular Case of Lactation,............................................61
Subscriptions Received,............................................192
Shelby Medical College,............................................760, 705
St. Louis Medical Society,..................137, 399, 609
Silver Sutures in Surgery,............................................507
Supra-Renal Capsules,............................................408
Surgical Excerpts-Calculus,............................................317

PAGE.
The Rights of Authors,............................................215
To Subscribers,............................................247
The Progressive Development of Physiological Ideas,............................................283
The American Medical Association, &c..........298
The Last New Medicine,............................................318
The Cincinnati Lancet and Observer,..............278
The Pacific Med. & Surg. Journal,..................444
The Pathology, &c., of Hemorrhage from Mucous Surfaces,............................................497
Total Ablation of the Inferior Maxillary. By T. Means,............................................643
The Hypophosphites,............................................694
The Excito-Secretory System,............................................117
Tubercular Pthisis,............................................733
The Great American Medical Regulator,.........126
Treatment of Asphyxia,............................................192
The Plantago Major,............................................192
The Medical Treatment of Insanity,.............. 28
The "Hotel Endemic" at Washington,........... 44
The Third Volume,............................................54
The late Meeting of the American Medical Association at Nashville,............................................57
Upon the Cure of Popliteal Aneurisms. By W. F. Westmoreland,............................................265
Varieties in Disease. By T. Pollard,................451
Valedictory Address,............................................530
Virginia Medical College,............................................704
Virginia Medical Journal,............................................706
Valedictory Address. By T. S. Powell,........... 65
Wynne's Case Book,............................................730

☞ The Second Volume of the Atlanta Medical Journal, neatly bound may be had of the Publishers, Messrs. G. P. Eddy & Co., at $2 50.

---

☞ The index of the Second Volume of the Journal is sent out with this number: any subscriber failing to receive it will please notify the editors.

The Medical Treatment of Insanity, ..... 28

Review of Nature and Art in the cure of Disease, ..... 33

The "Hotel Endemic" at Washington, ..... 44

Pork and Potatoes—their Influence on the Bodies and Minds of our People.... 49

### EDITORIAL AND MISCELLANEOUS.

The Third Volume, ..... 54

American Medical Association and Medical Education, ..... 55

The late meeting of the American Medical Association at Nashville ..... 57

Close of the Session, ..... 59

Fiske Medical Prize, ..... 59

Gelseminum Sempervirens in Gonorrhœa, ..... 60

New York State Lunatic Asylum, ..... 60

Consolidation of Medical Schools, ..... 60

Ergot in Menorrhagia, ..... 60

Preservation of Vaccine Virus, ..... 61

Singular case of Lactation, ..... 61

Iodide of Calomel, ..... 62

Dysentery, ..... 62

Interesting to Physicians, ..... 62

Electricity in the Suppression of the Lacteal Secretion ..... 63

Ergotine in Epidemic Diarrhœa, ..... 63

Arsenic and Stillingia in Diseases of the Skin, ..... 63

Meteorological Table, ..... 64

### TO CORRESPONDENTS.

☞ All Communications pertaining to the Editorial Department of the Journal, should be addressed to the Editors.

☞ All Communications, having reference to the Subscription, the Advertising or the Financial Department, should be addressed to G. P. EDDY & Co.

